# Speech Rhythm Facilitates Syntactic Ambiguity Resolution: ERP Evidence

**DOI:** 10.1371/journal.pone.0056000

**Published:** 2013-02-08

**Authors:** Maria Paula Roncaglia-Denissen, Maren Schmidt-Kassow, Sonja A. Kotz

**Affiliations:** 1 Max Planck Institute for Human Cognitive and Brain Sciences, Leipzig, Germany; 2 Institute of Medical Psychology, Goethe University Frankfurt, Frankfurt am Main, Germany; University Of Cambridge, United Kingdom

## Abstract

In the current event-related potential (ERP) study, we investigated how speech rhythm impacts speech segmentation and facilitates the resolution of syntactic ambiguities in auditory sentence processing. Participants listened to syntactically ambiguous German subject- and object-first sentences that were spoken with either regular or irregular speech rhythm. Rhythmicity was established by a constant metric pattern of three unstressed syllables between two stressed ones that created rhythmic groups of constant size. Accuracy rates in a comprehension task revealed that participants understood rhythmically regular sentences better than rhythmically irregular ones. Furthermore, the mean amplitude of the P600 component was reduced in response to object-first sentences only when embedded in rhythmically regular but not rhythmically irregular context. This P600 reduction indicates facilitated processing of sentence structure possibly due to a decrease in processing costs for the less-preferred structure (object-first). Our data suggest an early and continuous use of rhythm by the syntactic parser and support language processing models assuming an interactive and incremental use of linguistic information during language processing.

## Introduction

Over the past decades, several psycholinguistic studies have addressed the importance of prosody in sentence comprehension (e.g., [1]–[5]). It has been shown that prosody is used in early stages of sentence parsing (e.g., [4]–[6]) and that it can help to resolve structural ambiguity (e.g., [1]–[3]). In addition, appropriate prosody can be used as a local cue to facilitate syntactic processing or make it more difficult when inconsistent with syntactic structures (e.g., [1], [2], [4], [7]–[15]). Furthermore, prosody has been shown to influence several linguistic functions, such as phonology (e.g., [15]), semantics and pragmatics (e.g., [10], [16]–[20]), and syntax (e.g., [21], [22]).

Prosody can be understood as the acoustic features of spoken languages, such as duration, amplitude and fundamental frequency [23], manifested in at least two facets: intonation and rhythm. While intonation concerns the speaker-controlled pitch variation in course of an utterance, rhythm regards the temporal organization of the speech, allowing for segmentation of events in the utterance, i.e., sounds and pauses, and structuring them in a pattern of recurrence in time [24]–[27].

So far, studies investigating the importance of prosody to disambiguate syntactic structure have mainly addressed its intonational facet (e.g., [1]–[3], [7], [8], [13]). To our knowledge no study has specifically investigated the role of *rhythm* as a sentence segmentation cue to disambiguate syntactic structure and to facilitate sentence comprehension. Regarding the role of speech rhythm in auditory speech and language comprehension, previous studies suggest that listeners are sensitive to rhythmic regularity in speech (e.g., [28], [29]) that a word's metric property influences lexical access (e.g., [30]), interacts with semantics [20], [31] and with syntax (e.g., [15], [21], [32]).

However, speech rhythm should also be investigated as a broader phenomenon rather than just a local one during sentence processing. When speech rhythm operates, it not only organizes sounds into words, but also words into larger prosodic units [6], [17] as part of a prosodic hierarchy [33], [34], which may constitute units of perception [35]–[38]. Rhythm allows to segment relevant linguistic information, e.g., sounds, as speech flows, grouping it into meaningful linguistic units, e.g., words. These linguistic units may then be integrated with information from other linguistic domains, such as semantics and syntax, so comprehension is achieved [21], [26], [31], [39]. Given its significant contribution to speech organization, the role of rhythm should be investigated, not only when it operates as a local cue at the lexical level, but also when it serves as a sentence segmentation device, i.e., prior to and during the processing of syntactic complexity.

To our knowledge, there have been no studies investigating the role of rhythm as a sentence segmentation device during syntactic ambiguity resolution using the ERPs. ERPs are of great advantage while investigating unfolding language processes, such as the use of speech rhythm in sentences segmentation, because they capture the exact time course, in which these processes occur [40]. In this sense, the use of ERPs may contribute to a better understanding of ongoing linguistic processing, allowing to expand theories and models of language processing [14], [40].

So far, a few studies have used ERPs to investigate the role of prosodic breaks, as a local cue and influencing the syntactic parser during ambiguity processing (e.g., [14], [41]). In these studies, the ERP component Closure Positive Shift (CPS) was associated with the occurrence of prosodic breaks, while an enlarged N400 was elicited by the less-preferred syntactic structure, object-first sentences. This enlarged N400 was previously associated with difficulty in lexical integration (e.g., [42]–[44]), such as the encounter of an intransitive verb when a transitive one would be preferred (e.g., [9], [14]). In addition, an enlarged P600 elicited by object-first structures was found (e.g. [14], [41]), which was linked to the re-analysis of this less-preferred syntactic structure (e.g., [45]–[47]).

In the current study, we investigated the role of *rhythm* as a sentence segmentation cue, grouping words together in regular rhythmic chunks so as to facilitate the processing of syntactically ambiguous sentences. In previous experimental work, it has been suggested that the parser makes use of prosodic information, in our case rhythm, to create low-level syntactic structures, grouping words in “chunks” [5], [48], [49]. These chunks would remain unattached until enough morphosyntactic information is provided, reducing memory load, without forcing the listener to commit to a possibly wrong syntactic analysis. Our view is consistent with the existence of a prosodic representation available already during early stages of sentence processing (e.g., [4], [7], [8], [16], [22]) that interacts with the syntactic parser prior to, during, and after syntactic ambiguity is encountered [7], [8], [16].

Therefore, we presented participants with German sentences containing syntactic ambiguity, spoken in either regular or irregular rhythmic patterns. Rhythmic regularity was established by using one stressed syllable followed by three unstressed ones that created clitic groups (groups of grammatical words carrying one primary stress only [34]) of constant size.

In order to focus on syntactic re-analysis and avoid lexical integration difficulty, we used only transitive verbs (i.e., verbs requiring an accusative argument). In this sense, we expected to find a P600 response, which has been interpreted to indicate syntactic re-analysis of a less-preferred structure, i.e., object-first order (e.g., [14], [45], [47]).

By presenting ambiguous sentences in rhythmically regular context, we provide a reliable segmentation cue, namely stress patterns, creating rhythmic chunks. These rhythmic chunks operate clustering linguistic constituents, such as morphemes and grammatical words sharing one common primary stress (i.e., a clitic group; [33]). As a result of their acoustic salience, i.e., shared primary stress, these clusters constitute perceptual units in the speech stream. Perceptual units may guide the syntactic parser [6], [17], [35]–[38] when structures of greater syntactic complexity are encountered (i.e., object-first sentences), facilitating their processing.

It could be the case that rhythm facilitates the processing of both syntactic structures, i.e., subject-first and object-first order, however, its benefits should be more valuable and, therefore, more apparent during the processing of sentences with enhanced processing costs (i.e., object-first sentences), as in such cases, any facilitation cue can be used. Such facilitation should be confirmed by a significant reduction in the P600 mean amplitude response to object-first rhythmically regular sentences compared to the same structure in a rhythmically irregular context. Furthermore, behavioral results, such as higher accuracy rates and faster response times, should also be found for the less-preferred syntactic structure, i.e., object-first sentences, in rhythmically regular context in comparison to their rhythmically irregular counterparts.

## Methods

### Ethics Statement

This study was approved by the ethics committee of the University of Leipzig. All individuals in this study gave their written informed consent for data collection, use, and publication.

### Participants

Thirty-two participants (17 males; *M*_age_ = 25.59, *SD* = 2.53) participated in an initial rating study of the material, while twenty-four different participants (12 female; *M*_age_ = 26.33, *SD* = 1.97; all right-handed) took part in the EEG experiment. Participants from both studies were students of the University of Leipzig, native speakers of German, and were paid for their participation. None of the participants reported any neurological impairment or hearing deficit, and all had normal or corrected-to-normal vision.

### Material

Originally, we created 480 sentences using 60 transitive verbs (requiring an accusative complement combined with 120 different common and proper nouns. By using transitive verbs instead of intransitive ones (i.e., verbs requiring dative complements) we focused on sentence reanalysis (P600; [14], [47], [50]), avoiding responses to difficulties in lexical integration (N400; [42], [44]). Half of the sentences constituted experimental items, whereas the other half were filler sentences. Experimental sentences consisted of one main clause followed by a relative clause, i.e., the clause of interest, and were presented in a 2×2 design, with the factors *argument position* (subject-first *vs.* object-first order) and *rhythm* (irregular *vs.* regular rhythm). This resulted in sentence quadruplets, with each sentence corresponding to one of the four experimental conditions: subject-first rhythmically irregular, SFI; subject-first rhythmically regular, SFR; object-first rhythmically irregular, OFI; object-first rhythmically regular, OFR. Fillers and experimental sentences were between 17 and 19 syllables long (*M* = 17.1, *SD* = 0.36).

**Figure 1:**
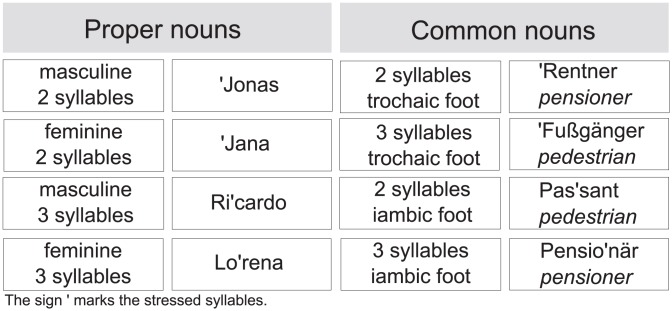
Examples of proper and common nouns used in the stimulus material.

Rhythmic regularity was established by a constant metric pattern of one stressed syllable followed by three unstressed ones, while rhythmic irregularity was achieved through the use of proper nouns of different syllable numbers, and common nouns that varied in terms of lexical stress and the number of syllables (for illustration of these properties, see Figure 1). Word frequency for common nouns was counterbalanced across the rhythmically regular and irregular sentence conditions and were not significantly different, z* = *0.13, *p*>0.1.

**Figure 2:**
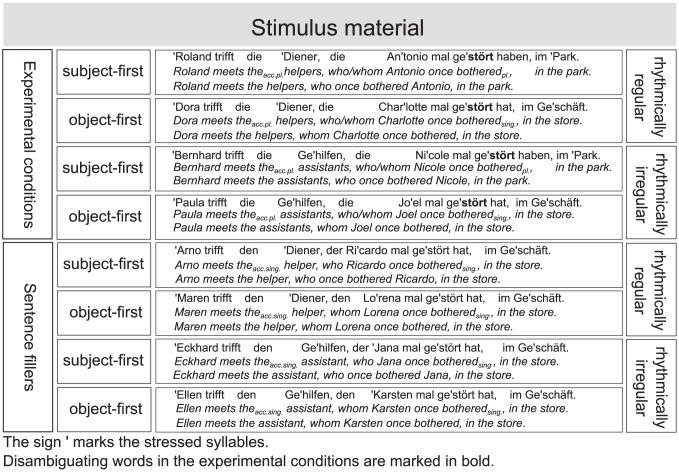
Exemplary sentence for each experimental condition and filler sentences.

The original 480 sentences were pseudo-randomized and arranged in 32 different written questionnaires to be rated by participants in terms of sentence content, according to a 7-point acceptability rating scale (1 = *unacceptable* and 7 = *highly acceptable*). Sentences with a mean rate of less than 4 points on the acceptability scale were removed from the stimulus material together with their experimental condition counterparts and matching fillers. This resulted in a total of 352 sentences (73.4% from the original sentences), i.e., 44 per condition, with corresponding fillers to be used as final stimulus material in the EEG experiment. The four experimental conditions, as well as their corresponding filler items, are presented in Figure 2.

These 352 final sentences were spoken by a German female professional speaker at a normal speech rate and digitally recorded via a computer with a 16-bit resolution and a sampling rate of 44.1 kHz. In order to prevent participants having access to any prosodic information other than speech rhythm, such as pitch contour variations, sentences were constructed with the application of a cross-splicing procedure.

#### Cross-splicing

The cross-splicing procedure, i.e., the procedure of replacing an existing sound with another one, was conducted separately for each sentence quadruplet (SFI, SFR, OFI and OFR). Stimuli cross-splicing was accomplished in four steps, using the software Praat (version 5.2.13).

Subject-first rhythmically irregular (SFI) sentences from each quadruplet were chosen as “standards”; i.e., their words were used as replacements for equivalent words in the remaining experimental conditions of the quadruplet. This was the case because SFI sentences present the preferred syntactic order in German, i.e., subject-first order, and their rhythm is natural (not experimentally manipulated). Because of this, we could create a more natural stimulus material which is also closer to natural speech. In a first step, the German plural relative pronoun (“die”/*the*) from the standard sentence (SFI) replaced its equivalents in the other conditions, i.e., SFR, OFI, OFR. Second, we utilized the segment immediately after the proper noun, containing the adverb and the participle of the main verb, from the standard sentence (SFI) to replace its equivalent in the other conditions (SFR, OFI, OFR). Third, the critical item, the auxiliary verb (“haben”*/have*), from the standard sentence (SFI) was used to replace its equivalent in its counterpart SFR sentence. Fourth, the same procedure as in step three was adopted, but this time, the auxiliary verb (hat/*has*) in the OFI sentence was used as a replacement for its equivalent in its counterpart OFR sentence. After applying the cross-splicing procedure, sentences were presented to 3 German native speakers and naïve listeners, who evaluated the naturalness of the sentences. None of the listeners reported hearing cuts, co-articulations or unnatural sounds in the sentences.

### Procedures

Participants were tested individually in a sound-attenuating booth, seated in a comfortable chair and requested to move as little as possible during the experiment. Participants performed a comprehension task, evaluating if the content of an auditorily presented sentence matched the content of a subsequently presented visual sentence. Prior to the experiment, participants received a short training session with 2 blocks of 16 sentences each (2 per condition and 8 equivalent fillers).

Each trial started with a red asterisk presented on the center of a black computer screen. After 1500 ms, the red asterisk was replaced by a white one and, at the same time, a sentence was presented via loudspeakers. With the offset of the auditory sentence, participants saw a written rephrased version of the previously heard relative clause. Participants were instructed to press the response keys of a button box as quickly and accurately as possible: with the “yes”-key if the content of the auditorily and visually presented sentences matched, or the “no”-key, if this were not the case. If, after 2.5 s participants failed to press any response key, a new trial was presented. The position of the correct-response key (left or right side) was counterbalanced across participants.

Sentences were pseudo-randomized and presented in 8 blocks of about 5.5 min each. Experimental blocks contained either rhythmically regular or irregular sentences and were presented in an alternating fashion. Sentences were presented in blocks of rhythmically regular or irregular sentence context which, in case of regularity, was hypothesized to provide a reliable segmentation cue during the disambiguation of syntactic structures. All participants started with a rhythmically irregular block to prevent possible facilitation/entrainment effects that may result from exposure to rhythmic regularity. After each context block, participants were offered a break. At the end of the session, participants were briefly asked about their perception of the stimulus material used, namely if they had perceived the use of rhythmic regularity in the spoken sentences. No participant reported having perceived rhythmic regularity in any of the presented sentences.

#### Electrophysiological Recordings

The EEG signal was recorded from 59 scalp sites by Ag/AgCl electrodes placed in an elastic cap (Electro Cap Inc, Eaton, OH, USA). Bipolar horizontal and vertical electro-occulograms (EOG) were recorded to allow for eye artifact correction. Electrodes were online re-referenced to the left mastoid and offline re-referenced to averaged left and right mastoids. Recording impedance was kept below 5 kΏ. EEG and EOG signals were recorded with a sample frequency of 500 Hz, using an anti-aliasing filter of 140 Hz. Trials affected by artifacts, such as electrode drifting, amplifier blocking and muscular artifact, were excluded from analysis (*M* = 4.78%, *SD* = 6.23), while trials containing eye movements were individually corrected, using an algorithm based on saccade and blink prototypes [51]. Trials were averaged separately per condition, i.e., SFI, OFI, SFR and OFR, and per participant (subject-average), and across all participants (grand average). Chosen epochs ranged from the onset of the critical item (i.e., the auxiliary verb and the disambiguating word; “haben”*/have* and “hat”/*has*) to 900 ms after its offset (i.e., at the onset of the visually presented sentence), and were calculated with a baseline of −200 to 0 ms. Further, all incorrectly answered trials were excluded from data analysis (*M* = 9.02%, *SD* = 10.04). For graphical display only, data were filtered off-line using a 7 Hz low pass filter.

#### Statistical Analysis

For accuracy rates (correct *vs.* incorrect responses) a logistic regression analysis was conducted using *argument position* (subject-first *vs.* object-first order) and *rhythm* (regular vs. irregular rhythm) as predictors.

For the reaction times analysis, a repeated-measures analysis of variance (ANOVA) was conducted using the two experimental factors *argument position* and *rhythm* as within-subject factors. In addition, as rhythmically regular sentences contained, on average, significantly less syllables (*M* = 9.23, *SD* = 0.50) than their rhythmically irregular counterparts (*M* = 9.74, *SD* = 0.972), z = 5.56, *p*<0.01, for *reaction times* analysis the *number of syllables* was used as covariate.

For the ERP data analysis, the time window ranging from 350 to 550 ms was chosen based on visual inspection and previous studies [47], [50], [52], [53]. In these studies an earlier than the classical positivity (P600) was elicited during the processing of case ambiguous subject-object relative clauses. It has been suggested that case ambiguous sentences, i.e., subject-first *vs.* object-first order, lead to a less severe Garden Path [47] for structural reasons [54] as well as for lower processing costs [55]. Consequently, the early latency in the positive response would result from the ease of reanalyzing a case ambiguous sentence [55]. However, some of the previous research also reported a late positivity together with an early one [47], [52]. The combined elicitation of two positivities may result from a more complex experimental setting, i.e. half of the sentences have to disambiguated at the final auxiliary verb (similarly to studies encountering an early positivity) and the other half at an earlier point of the sentence (noun phrase). Thus, it has been suggested that the late positivity may account for a secondary verification of structural adequacy, and more likely occurring in experimental settings containing different types of case ambiguous sentences.

Furthermore, a repeated-measures ANOVA quantifying the mean amplitude data was conducted using the two experimental factors *argument position* (subject-first *vs.* object-first order) and *rhythm* (regular *vs.* irregular rhythm), and two topographical factors *region* (anterior *vs.* posterior region) and *hemisphere (*left *vs.* right hemisphere) as within-subject factors. *Region* and *hemisphere* comprised four regions of interest (ROIs), constituted by 6 electrodes each: *left anterior* (F1, F3, F5, FC1, FC3, FC5), *right anterior* (F2, F4. F6, FC2, FC4, FC6), *left posterior* (CP1, CP3, CP5, P1, P3, P5) and *right posterior* (CP2, CP4, CP6, P2, P4, P6). To focus on main results, only significant main effects and interactions of critical factors, namely *argument position* (subject-first *vs.* object-first order) and *rhythm* (irregular *vs.* regular rhythm), are reported.

## Results

### Behavioral Results

#### Accuracy rates

**Figure 3:**
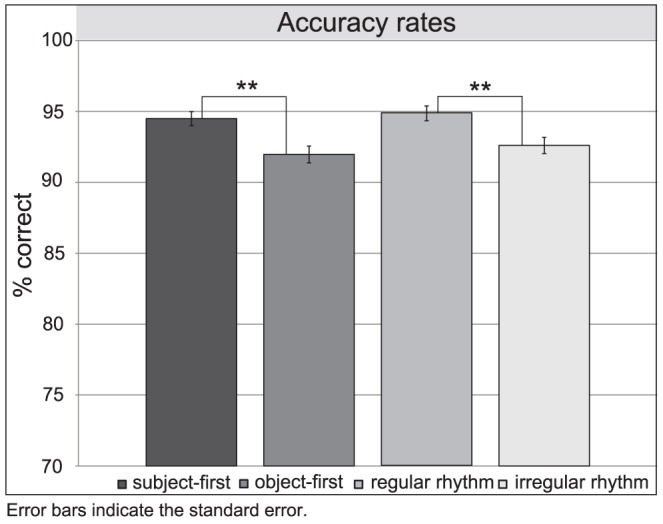
Accuracy rates for *argument position* and *rhythm* in the comprehension task.

Overall correct response rates were above 90% (*M_SFI_* = 93.95%, *SD* = 23.84, *M_OFI_* = 91.25%, *SD* = 28.25, *M_SFR_* = 95.12%, *SD* = 21.54 and *M_OFR_* = 92.69%, *SD* = 26.02). The full logistic model was significant, indicating that the experimental factors significantly predict participants' scores (X^2^ = 14.99, p<0.001 with df = 2). The Wald criterion revealed that argument position (X^2^ = 10.14, *p*<0.01) and rhythm (X^2^ = 4.88, *p*<0.05) made a significant contribution to prediction for participants' scores (p<.001). A follow-up analysis indicates that participants had higher scores for subject-first sentences (*M = *94.54%, *SD* = 22.72) than for object-first order (*M = *91.97%, *SD* = 27.16) and for rhythmically regular sentences (*M = *93.91%, *SD* = 23.99) in comparison to rhythmically irregular ones (*M = *92.61%, *SD* = 27.16). Table 1 presents the logistic regression analysis of participants' accuracy rates and Figure 3 the accuracy rates for *argument position* and *rhythm* in the comprehension task.

**Table 1:** Logistic regression analysis of participants' accuracy rates.

Predictor	B	*SE* β	*Wald's X^2^*	*df*	*P*	*e^β^ (odds ratio)*
Constant	-2.6869	0.1093	604.6390	1	*<0.001*	NA
Argument position (subject-first = 0, object-first order = 1)	0.3952	0.1232	10.1383	1	*0.0015*	1.4850
Rhythm (irregular = 0, regular = 1)	−0.2722	0.1241	4.8825	1	*0.0271*	0.7620

Test	*X^2^*	*df*	*P*
Overall model evaluation			
Likelihood ratio test	15.2171	2	0.0005
Score test	15.1416	2	0.0005
Wald test	14.9871	2	0.0006
Goodness-of-fit Test			
Hosmer & Lemeshow	0.1270	2	0.9385

Kendall's Tau-a = 0.0170; Goodman-Kruskal Gamma = 0.1750; Somers's Dxy = 0.1320; c-statistic = 56.60%. For statistical precision, all statistics here reported use 4 decimal places. NA = not applicable.

#### Reaction times

**Figure 4:**
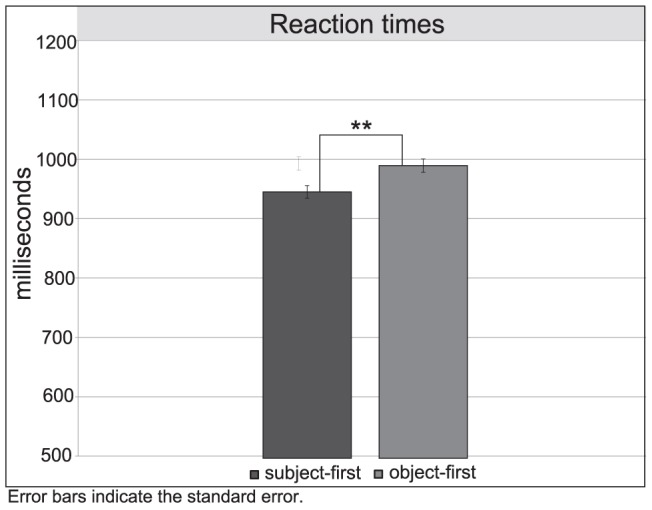
Reaction times for subject-first and object-first sentences in the comprehension task.

Overall participants' reaction times were faster than 1100 ms (*M_SFI_* = 989.82 ms, *SD* = 487.63, *M_OFI_* = 1044.86 ms, *SD* = 514.80, *M_SFR_* = 907.79 ms, *SD* = 451.86 and *M_OFR_* = 926.35 ms, *SD* = 446.88). Results revealed a significant main effect of *argument position*, *F*(3,75) = 3.14, *p*<0.05, with faster responses for subject-first (*M = *963 ms, *SD* = 472) than for object-first order (*M = *1004 ms, *SD* = 484). Mean reaction times for subject-first and object-first sentences are presented in Figure 4. Contrary to what we initially expected, no significant effect of *rhythm* and no interaction between the two experimental factors were found.

### ERP Data

**Figure 5:**
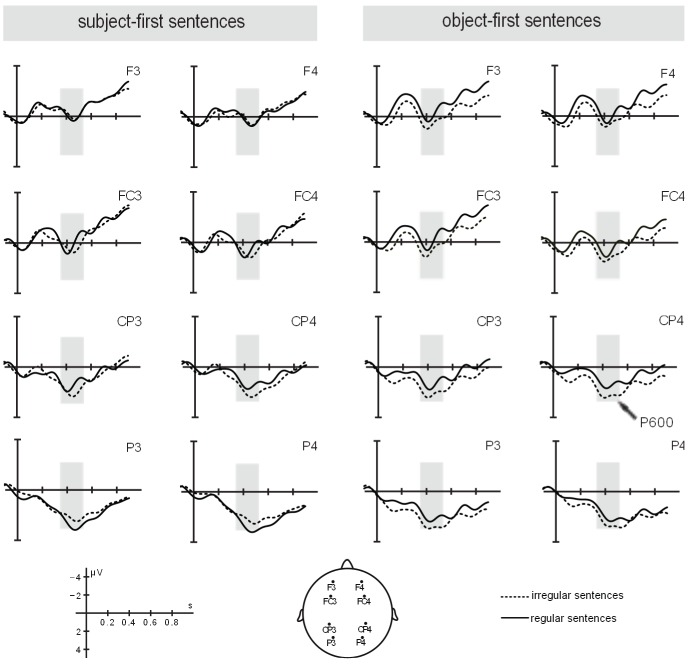
ERP responses for experimental conditions in the time window of interest.

A repeated-measures ANOVA revealed a significant interaction between *argument position* and *rhythm, F*(1, 23) = 6.66, *p*<0.05. When resolving this interaction for *argument position*, a significant main effect of *rhythm* was found for object-first sentences only, *F*(1, 23) = 4.36, *p*<0.05, with a smaller P600 mean amplitude in rhythmically regular sentences (*M = *1.10 µV, *SD* = 2.95) than in their rhythmically irregular counterparts (*M = *2.04 µV, *SD* = 2.06), corroborating our initial hypothesis. For subject-first sentences, the analysis did not yield statistically significant differences between rhythmically regular and irregular sentences, *p*>0.1; also in line with what we initially expected. No further significant interactions or main effects for the critical factors were found. Figure 5 depicts ERP responses for experimental conditions in the time window of interest.

## General Discussion

In the current work, we utilized ERPs as well as behavioral measures to investigate the impact of speech rhythm as a segmentation cue during the processing of sentential syntactic ambiguity. We presented participants with syntactically ambiguous sentences embedded in regular and irregular rhythmic contexts. By providing participants with a rhythmically regular context, we expected to see a reduction of processing costs for the less-preferred syntactic structure, i.e., object-first sentences if regular rhythm works as a sentence segmentation device.

Our results partially corroborate the proposition that regular rhythm facilitates the processing of the less-preferred syntactic structure, i.e., object-first sentences. On the one hand, behavioral results, confirm rhythmic facilitation of overall accuracy rates, but independent of sentence structure type. On the other hand, in line with our hypothesis, ERP data confirm a significant rhythmic facilitation effect for the less-preferred syntactic order only (i.e., object-first sentences). This rhythmic facilitation effect is revealed by a significantly reduced P600 mean amplitude response to object-first sentences in rhythmically regular context only.

One possible explanation why behavioral results not to depict an interaction between rhythm and sentence structure type may be due to the fact that behavioral measures may only capture the outcome of the syntactic disambiguation, at the end of sentence processing. If an interaction of *rhythm* and *argument position* occurs as the sentence unfolds, then behavioral measures may not be sensitive enough to reveal such an interaction. In order to depict the complexity of an ongoing process (i.e., the use of rhythm as a sentence segmentation cue), online measures, such as ERPs, may be better suited for detecting the more immediate effects of rhythm. An alternative explanation for the differences between the behavioral and the ERP results could be based on participants' qualitatively different online and task specific responses. While behavioral measure may reflect the decision of whether the auditory and the visual rephrased sentence are the same, ERPs may reflect the response to the encountered ambiguity. Thus different task and non-task related aspects may be reflected in the two measures.

Yet, one may also argue that the use of a constant metric pattern does not occur naturally in spontaneous speech, and therefore our result reflects an artificial consequence of our manipulation. However, this reasoning seems unlikely, because a post-experimental debriefing revealed that participants did not perceive rhythmic regularity in any of the sentences they listened to. This suggests that even though rhythmicity was manipulated, this was done in a natural not obvious (i.e. as spoken by a metronome) fashion.

Our findings provide new evidence of how prosodic information may affect the disambiguation of syntactic structure during sentence processing. First, while previous research has focused exclusively on the role of *intonation* [6], [7], [10], [15], [16], [56] on syntactic processing, this is the first study to address the temporal nature of prosody, namely *rhythm*, during the disambiguation of syntactic structures. Second, previous research has investigated the role of intonation, i.e., prosodic breaks, as a local cue which may be used to facilitate syntactic processing [9], [14], [41]. Here, we addressed the role of rhythm during ongoing sentence processing, that is even before encountering syntactic ambiguity. Hence we investigated a broader scope of how rhythm operates as a segmentation cue during online sentence processing.

Our work is consistent with the idea of an existing prosodic representation available already in early stages of language processing, which interacts with the syntactic parser, guiding it through the processing of syntactic constituents [7], [8], [16], [48], [49]. Further, our work is based on the idea that prosodic units, in our case rhythmic groups, constitute perceptual units [36], [38], [57], which in turn operate as processing units [6], [17], reducing the memory load and facilitating language processing [16], [48], [49]. Thus, in the current work, we provided participants with a prosodic representations based on rhythmic regularity, which created a reliable segmentation context for the unfolding sentence, reducing the processing costs of the less-preferred syntactic structure, i.e., object-first sentences.

The importance of rhythm for speech segmentation in first language acquisition has already been shown. Studies conducted with preverbal infants reveal that infants rely on rhythmic information from their native language in order to segment speech and encode their first words [58]–[60]. During this process, they appear to refine their ability to discriminate rhythmic information in their native language [61], [62], encoding rhythm as phonological information [63].

Once encoded, rhythm helps the listener to organize sounds and pauses in spoken language in form of a prosodic hierarchy that helps to structure an utterance at several levels and various points in time [33]. Thus, rhythm organizes sounds and pauses in the speech flow into words that can be grouped together in a clitic group (a group of grammatical words presenting one common primary stress only). Clitic groups, in turn, can be combined to create phonological phrases (i.e., clusters of clitic groups), which can be integrated into intonational phrases (a linguistic segment with one complete intonational contour, [34]).

Our results are in line with previous studies suggesting that prosodic units may act as processing units, guiding the syntactic parser through the speech stream [6], [7]. Our research corroborates previous findings revealing that prosody, in our case rhythm, facilitates information processing when larger information chunks are provided [64], [65]. Thus, keeping all sentential cues constant (i.e., phonological, semantic, syntactic, pragmatic and intonational) rhythm may become a salient segmentation cue, which, in turn, may increase efficiency in sentence processing. Hence, rhythm is used to guide the syntactic parser through the processing of larger information units.

One could also argue that rhythm operates as a sentence segmentation cue regardless of which syntactic structure is being processed. However, its benefit may only become apparent when syntactic difficulty increases. Therefore, future studies should investigate the role of rhythm in a broader range of syntactic complexities during sentence processing.

In this sense, the two prosodic facets, i.e., *intonation* and *rhythm*, help to facilitate syntactic processing though in a different manner. On the one hand, *intonation* may provide complementary information to be integrated by the syntactic parser when syntactic ambiguity occurs, and thus facilitates processing [4], [7], [9], [14], [15]. On the other hand, as our study reveals, *rhythmic* regularity may already impact sentence segmentation prior to ambiguity resolution, thus facilitating information processing, and consequently reducing the overall processing costs for syntactically ambiguous sentences. Our results provide evidence of the early and continuous use of rhythm by the syntactic parser. This evidence is consistent with language processing models assuming an interactive and incremental use of linguistic information during sentence processing [6]–[8], [16], [17], [48], [49].

In view of these results, some questions remain. Is facilitation by means of rhythmic regularity a language-dependent or language-independent phenomenon? Some studies have shown that the perception of speech rhythm and its use as a word segmentation cue is language dependent [66]–[68]. Other studies investigating the cognitive ability of listeners have provided evidence that rhythm in its function of grouping elements together facilitates syllable and word recall independent of the rhythmic class of a language [69]–[71]. Therefore, even though rhythm as a device to segment the speech stream may be language specific, perhaps its use beyond the word level, i.e. when grouping words together, may not be.

If the use of rhythm in grouping organizing the speech stream is a universal and language-independent property, second language (L2) learners may also use rhythmic regularity in the L2 to facilitate syntactic processing. Thus, further investigations regarding the perception and the use of rhythmic regularity as a sentence segmentation cue in the context of L2 processing are called for. Such investigations should shed more light on the perception and use of rhythm in a broader sense, i.e., beyond the level of word segmentation, as a potential cross-linguistic or language-dependent phenomenon.

### Conclusion

In the current work we investigated the role of rhythm as a sentence segmentation cue during the disambiguation of syntactic structures. Rhythmic regularity was achieved by the use of a constant metric pattern of three unstressed syllables between two stressed ones. Accuracy rates suggest that rhythmic regularity facilitates overall sentence comprehension. ERP results indicate a reduction of the P600 mean amplitude in response to the less-preferred syntactic structure, i.e., object-first sentences, in rhythmically regular context only. Our results suggest that rhythm may be used as a reliable sentence segmentation cue, facilitating the processing of non-preferred syntactic structures, i.e., object-first sentences, and improving sentence comprehension.

## Supporting Information

Audio S1 **Example of experimental sentence for rhythmically irregular subject-first order.**
(WAV)

Audio S2 **Example of experimental sentence for rhythmically irregular object-first order.**
(WAV)

Audio S3 **Example of experimental sentence for rhythmically regular subject-first order.**
(WAV)

Audio S4 **Example of experimental sentence for rhythmically regular object-first order.**
(WAV)

Audio S5 **Example of filler sentence for rhythmically irregular subject-first order.**
(WAV)

Audio S6 **Example of filler sentence for rhythmically irregular object-first order.**
(WAV)

Audio S7 **Example of filler sentence for rhythmically regular subject-first order.**
(WAV)

Audio S8 **Example of filler sentence for rhythmically regular object-first order.**
(WAV)
